# Where there is no hospital: improving the notification of community deaths

**DOI:** 10.1186/s12916-020-01524-x

**Published:** 2020-03-09

**Authors:** Tim Adair, Megha Rajasekhar, Khin Sandar Bo, John Hart, Viola Kwa, Md. Ashfaqul Amin Mukut, Matthew Reeve, Nicola Richards, Margarita Ronderos-Torres, Don de Savigny, Daniel Cobos Muñoz, Alan D. Lopez

**Affiliations:** 1 Melbourne School of Population and Global Health, The University of Melbourne, Carlton, VIC 3053 Australia; 2 Cabinet Division, Government of Bangladesh, Dhaka, Bangladesh; 3 Ministry of Health and Social Protection, Bogota, Colombia; 4 Swiss Tropical and Public Health Institute, Basel, Switzerland; 5 University of Basel Institute, Basel, Switzerland

**Keywords:** Cause of death, Civil registration and vital statistics, Community, Death notification, Death registration, Innovation, Mortality, Verbal autopsy

## Abstract

**Background:**

Globally, an estimated two-thirds of all deaths occur in the community, the majority of which are not attended by a physician and remain unregistered. Identifying and registering these deaths in civil registration and vital statistics (CRVS) systems, and ascertaining the cause of death, is thus a critical challenge to ensure that policy benefits from reliable evidence on mortality levels and patterns in populations. In contrast to traditional processes for registration, death notification can be faster and more efficient at informing responsible government agencies about the event and at triggering a verbal autopsy for ascertaining cause of death. Thus, innovative approaches to death notification, tailored to suit the setting, can improve the availability and quality of information on community deaths in CRVS systems.

**Improving the notification of community deaths:**

Here, we present case studies in four countries (Bangladesh, Colombia, Myanmar and Papua New Guinea) that were part of the initial phases of the Bloomberg Data for Health Initiative at the University of Melbourne, each of which faces unique challenges to community death registration. The approaches taken promote improved notification of community deaths through a combination of interventions, including integration with the health sector, using various notifying agents and methods, and the application of information and communication technologies. One key factor for success has been the smoothing of processes linking notification, registration and initiation of a verbal autopsy interview. The processes implemented champion more active notification systems in relation to the passive systems commonly in place in these countries.

**Conclusions:**

The case studies demonstrate the significant potential for improving death reporting through the implementation of notification practices tailored to a country’s specific circumstances, including geography, cultural factors, structure of the existing CRVS system, and available human, information and communication technology resources. Strategic deployment of some, or all, of these innovations can result in rapid improvements to death notification systems and should be trialled in other settings.

## Background

Reliable evidence on the level and causes of the estimated two-thirds of global deaths that occur in the community is poor [1], with less than half of these deaths ever registered in civil registration and vital statistics (CRVS) systems [2, 3]. Even when community deaths are registered, information on the underlying cause of death (COD) is generally of poor quality due to both the lack of physicians to perform medical certification and poor certification practices [4]. A primary reason for the meagre state of policy-relevant evidence on community deaths is the lack of reliable means for the initial notification of the occurrence of the death; this is in contrast to deaths that occur in facilities, which generally have a formal system to notify a death event and medically certify its cause using internationally agreed standards. Initial notification of the occurrence of death is a crucial first step in the process towards eventual registration (Table 1). Additionally, improvements in both notification and registration practices are essential to provide better evidence to inform population health policy and planning. Improving global practices for the notification of community deaths is therefore an essential first step in providing better evidence to inform population health policy and planning to prevent them.

**Table 1:** Key terms [5]

Notification. The capture and onward transmission of minimum essential information on the fact of birth or death by a designated agent or official of the CRVS system, using an authorised notification form (paper or electronic), with that transmission of information being sufficient to support eventual registration and certification of the vital event.	
Registration. The act of formally registering an event at a civil registration office. At this point, the details of the event are recorded in the official civil register by the registrar.	

Death notification is usually performed by a designated agent or official, who generally provides the minimum essential information to initiate further government processes (Table 2). Importantly, the act of notification of a community death can, and should, trigger a household visit by a community health worker or other trained personnel to carry out a verbal autopsy (VA) interview with the family, thus greatly increasing the policy relevance of the information by providing essential COD data. The process of death notification is generally much simpler than registration (which requires multiple additional steps to complete) as well as being easier and faster, particularly with the availability of new communication technologies.

**Table 2:** Minimum essential information required for death notification [6]

The following information is commonly the minimum required for notification of a death, regardless of the mode of notification. *Numbers in brackets relate to the topics and themes to be investigated for vital statistics purposes through the CRVS system, as defined by the UN Principles and Recommendations for a Vital Statistics System.*	
1. Information about the deceased	
a) Full name	
b) Date of birth or age at death (*11*)	
c) Sex (*12*)	
d) Address (*6*)	
2. Information about the death event	
a) Date of death (*1*)	
b) Place of death (*3*)	
c) Cause of death if medically attended, or mode of death (*41*)	
3. Information about the declarant/informant	
a) Full name	
b) Address and phone number	
c) Relationship to the deceased	

While the complete registration of deaths with full accompanying information on the decedent should be the goal for any routine mortality surveillance system, notification of the fact of death, along with essential minimum demographic information, can serve several policy needs, particularly if the notification is linked to a procedure for a VA to ascertain probable COD. In such a system, all notified deaths would be registered, as is required for subsequent civil procedures and, in many cases, burial. This is the case, for example, in Kenya and South Africa, where notification and registration are linked and need to be completed in order to obtain a burial permit [7].

In most low- to middle-income countries (LMICs) the increasing accessibility of information and communication technologies (ICT) can be harnessed to facilitate innovative methods for the notification of community deaths. In Uganda and Mozambique, for example, community notifiers use a mobile phone to send death (and birth) notifications to a central server, which is verified and registered by registration officers [8, 9]. In Namibia, the government has introduced an e-death reporting system that allows social services and mortuaries, which are often the first point of contact with the deceased, to notify and verify the identity of the deceased [10]. In Sierra Leone, during the Ebola epidemic of 2014–2015, a national alert system was established in the form of a toll-free phone number to detect new cases promptly, which is now in the process of being transformed into a routine death reporting system [11].

There is, however, less evidence as to how death notification can be sustainably linked to local community structures, such as health clinics and aid posts, to ascertain the COD. Determining the underlying cause for all deaths is vital for a country’s public health planning and social development agenda. This is reflected in the United Nations Sustainable Development Goals, which have 17 indicators requiring COD data [12]. A notification system that automatically prompts the conduct of a VA for deaths occurring in the community could, in principle, provide governments, especially ministries of health, with essential health intelligence on the leading causes of death in rural populations; this could, for instance, ensure that policies to adequately address the growing burden of non-communicable diseases in LMICs are based on local, reliable and timely evidence.

### Examining community death notification practices

Under the Bloomberg Philanthropies Data for Health (D4H) Initiative, 16 countries undertook a structured mapping exercise to identify and map current procedures, responsibilities and data flows for their CRVS systems [13]. This revealed substantial variation between countries with regards to existing processes for the notification of community deaths, with many lacking a clear, structured process for data consolidation and transmission [6]. Only around half of the countries had any identifiable process for notification of community deaths and a specific form for doing so. Across countries, a range of individuals could act as notification agents; in many countries, multiple systems captured information about the death, which was usually not integrated into a central record. Importantly, only five (less than one-third) of the countries had a system for active surveillance of community deaths, with the rest relying on ‘passive’ processes of notification, usually by family members (Table 3).

**Table 3:** Passive and active registration systems

‘Passive’ notification refers to the conventional notification practice for community deaths of waiting for the family to declare the event to a local authority to start the CRVS process, which is why so few community deaths are ever registered.	
‘Active’ notification, on the other hand, refers to the process whereby an agent of the CRVS or health system actively seeks out community deaths by managing and visiting community key informants or others, such as those who issue burial permits, and capturing the required information for notification or visiting the household to do so through a VA process.	

A conclusion from the mapping exercise was that countries required tailored approaches to strengthen and develop systems to suit their specific circumstances and available resources. To illustrate the innovative methods of improving death (and birth) notifications in resource-poor settings, we present case studies of four D4H partner countries: Bangladesh, Colombia, Myanmar and Papua New Guinea (PNG). The approaches taken demonstrate the ingenuity and flexibility required to address the complex challenges faced by these countries and highlight the significant potential that improvements to death notification systems can have to rapidly strengthen the evidence base of community deaths and their causes for policy and planning.

### Bangladesh

Less than 10% of deaths are registered in a timely manner in Bangladesh, despite it being mandatory [14]. A key reason for low registration is the passive notification system, whereby the family is required to collect documents showing proof of death from local authorities and then apply for death registration at a local registry office. As part of the D4H Initiative, a trial of an active method for notification of deaths (and births) in the community was conducted in the Kaliganj subdistrict of Gazipur, with the aim of improving rates of birth and death registration and identifying cause of death through VA. Originally named the ‘Kaliganj model’, the trial was expanded to the Kapasia subdistrict in 2017. For comparison, data from the Savar subdistrict of Dhaka were used as a control setting with no intervention.

The intervention involves community health workers using their routine household visits and regular ongoing Extended Programme on Immunisation services to identify deaths that occur in each local area under their mandate, assisting relatives of the deceased to complete the paper death registration application form. The form is then submitted to the health worker’s supervisor, who submits it to the local registry office where the ‘Union Parishad Chairman’ verifies the application, uploads the data into the online system and prepares the death certificate for the family. This linkage of notification and registration means the family only needs to take minimal action for a community death to be registered, compared with the passive registration system.

**Fig. 1:**
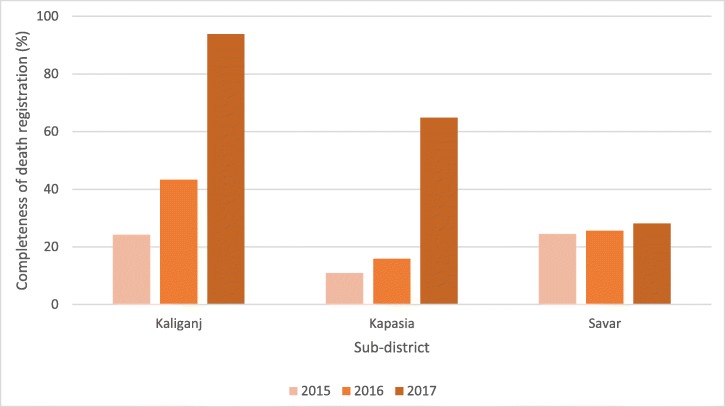
Comparison of completeness of death registration within 1 year for intervention (Kaliganj and Kapasia) and non-intervention (Savar) subdistricts, 2015–2017, Bangladesh

In Kaliganj, from September 2015–August 2016 (before intervention) to September 2016–August 2017 (after intervention), the percentage of all deaths that were registered within 1 year increased from 24 to 94% (Fig. 1) [15]. In Kapasia, from September 2016–August 2017 (before intervention) to the 3-month period of September–December 2017 (after intervention), the percentage of all deaths that were registered within 1 year increased from 11 to 65%. These results are impressive when compared with Savar, where this intervention was not introduced, and the percentage of all deaths that were registered within 1 year barely increased from 25% in 2016 to 28% in 2017.

An evaluation of the pilot intervention identified several factors that led to the success of the Kaliganj model, including effective sensitisation and training of field staff, resulting in high motivation, and engagement of community health workers who were already embedded in local communities and were able to assist in following-up the registration process with the Union Council (local registry office) [14]. A key benefit of the Kaliganj model is that it also identifies deaths for subsequent VA, thus addressing the significant gap in knowledge about the causes of community deaths in Bangladesh. The Kaliganj model for death notification provides an effective means of utilising existing health staff to improve information on community mortality patterns.

### Colombia

Colombia has a relatively well functioning vital registration system [3]. However, an estimated 15–20% of deaths are still not registered and occur in remote, rural or ethnic minority communities with little or no access to health facilities ([16], and personal communication with the National Administrative Department of Statistics (DANE), Colombia). An active death notification system, named ‘Colombia Rural Vital’, was trialled in 2018 to detect, report and ascertain COD (using VA) in 14 municipalities with a total population of 472,000. These municipalities were chosen based on their low crude mortality rate (as an indication of low completeness of death registration), remoteness and the presence of ethnic minorities.

Led by the Ministry of Health and local health authorities, and supported by D4H, the system used mobile phone text messages for initial reports of a death and a cloud-based database to compile the data. The strategy engaged a variety of community members for notification, including community health workers, religious leaders, police officers and traditional birth attendants. The following reporting methods were employed: (1) a free short messenger service (SMS) message sent by community leaders from rural or ethnic communities, containing minimum essential information about the event; (2) police and civil registry offices conveying known deaths to local health authorities; (3) administrators of community cemeteries in rural areas recording and sending an SMS for each death; and (4) paper reporting forms for communities where the telephone signal coverage is poor. The SMS messages automatically populated an online platform and alerted the local health authority, community health coordinator and surveillance officers about the death.

Following receipt of either the SMS or paper forms, a nurse coordinator or surveillance officer assigned the case to an auxiliary nurse or health promoter who validated the information and checked whether the death was already included in the national CRVS database. For each death, the health worker visited the family to conduct a VA interview using a tablet and uploaded the data to a cloud database.

As of February 2019, 279 deaths from 2017 and 2018, which had not been previously included in the national mortality database (personal communication DANE), were identified in the 14 municipalities through the different reporting methods. Of these deaths, a VA had been performed for 149, with a COD determined for 98% of them. The newly identified deaths meant an 11% increase in reported mortality (based on total number of deaths before and after the intervention) in 2017, a 10% increase in 2018, and an overall increase in reported rural population mortality of 16 and 14% for 2017 and 2018, respectively.

The ‘Colombia Rural Vital’ strategy has been effective for both the local health authorities and communities in better identifying the true level and pattern of mortality in these underserved populations, thus providing the evidence for government policies and programmes to focus attention and funding on appropriate interventions. The strategy has now been adopted as a part of the routine extramural activities of local hospitals within the 14 municipalities, and 2 of the participating local health authorities (covering 9 municipalities) have allocated resources to ensure the continuity of technical assistance in 2019.

### Myanmar

In Myanmar, it is estimated that the CRVS system registers about half of all deaths. There is an established health infrastructure in the country, the frontline of which is formed by basic health staff (BHS), including 30,000 health assistants, midwives, public health supervisors and lady health visitors that service the predominantly (70%) rural population [17]. Only about 16% of the estimated 400,000 deaths that occur each year take place in hospitals and receive a medically certified COD, with the remaining deaths occurring in the community, typically with a poor-quality COD assigned, or none at all [18]. Prior to the D4H Initiative, notification of community deaths was facilitated by the BHS, who were responsible for two separate paper-based methods to report the same event to both the Health Management and Information System of the Department of Public Health and, separately, to the national CRVS system maintained by the Central Statistical Organisation (CSO).

From April 2017 to March 2019, a pilot death notification intervention implemented across 42 townships (covering 8.2 million people, approximately 15% of the national population) led to a significant increase in the number of reported deaths when coupled with the introduction of VA. Following a death in the community, the village health administrator or volunteer informally notified the BHS of the event. The BHS used an electronic tablet to register the death and perform a VA at the home of the deceased. Basic notification information, including the most probable COD, was automatically sent from the tablet to a server based at the CSO. Data verification and analysis was then conducted by CSO staff.

**Fig. 2:**
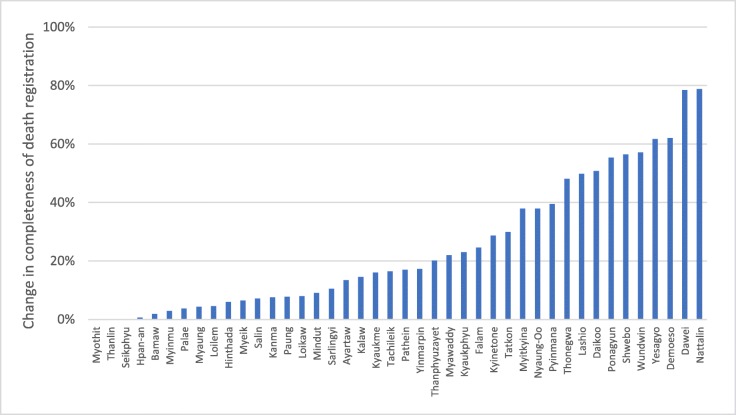
Percentage point change in completeness of death registration between 2016 and 2018 in 42 intervention townships, Myanmar

Importantly, as a result of the intervention, representative information on the causes of community deaths in Myanmar became available for the first time. Death registration in the 42 townships increased, on average, by 75% (from 38 to 66%) since 2016, with some townships increasing death registration from less than 5% to more than 80% (Fig. 2). Qualitative analysis suggested that the BHS were able to integrate the reporting responsibilities into their routine tasks and did not find the workload burdensome [19]. As part of ongoing improvements, it is planned that the process of birth and death notification will be upgraded to a digital system; additionally, the use of tablets for reporting births and deaths in the community has now been incorporated into the training programme of BHS.

### Papua New Guinea

CRVS processes in PNG require significant development, with only between 5 and 7% of all deaths registered [20]. Notification has always been a passive system in PNG; however, with the drafting of a new Civil Registration Bill, notification procedures are expected to become more active.

As part of the D4H Initiative, the use of different notifying agents and either paper or electronic notification processes were trialled in three districts in three separate provinces (Milne Bay, West New Britain and Western Highlands), all with varying geographies and local government structures. These three provinces had already started to use the new electronic National Health Information System to capture deaths in health facilities but community deaths continued to be unrecorded. In each district, the underlying strategy was for an initial report of a death to be delivered to the health centre either electronically or as a paper-based form, then for the health centre to verify the data, complete an electronic notification form and conduct a VA, finally uploading the data to a central server for consolidation and review at the National Department of Health in Port Moresby.

In three subdistrict areas of Milne Bay, known as Local Level Governments (LLGs), mobile phones were distributed to ward recorders to send an SMS with basic notification information on community deaths to their nearest health centre. To compare SMS to paper methods in the same district, paper forms were distributed to ward recorders for completion and delivery to the health centre in the remaining three rural LLGs in Milne Bay. In West New Britain, due to better transport links but poor telecom networks, paper forms were used, which were distributed to ward recorders in all LLGs. In Western Highlands, paper forms were distributed to village health volunteers (who act as notifying agents). Field coordinators employed at each site supported the notifying agents and, in areas where the literacy level of volunteers was low (such as the Western Highlands), facilitated the completion of paper forms and transfer of data from communities to health centres.

Both the paper and SMS strategies involved the health centres arranging visits to the households following initial notification, health workers verifying the information, completing electronic notification forms and a VA, and uploading of the data to a central server for review at the National Department of Health. The strategies acknowledge the issue of data security of electronic transmission of information. The SMS of the initial report of the death only included limited information of name and ward of residence, and therefore had limited potential data security issues. The more detailed information on the electronic notification form collected by the local health centre was transmitted using secure ODK software. The importance of data security issues is also reflected by the new Civil Registration Bill and Regulations, currently being drafted, that will facilitate transmission of notifications via mobile phone or paper methods.

**Fig. 3:**
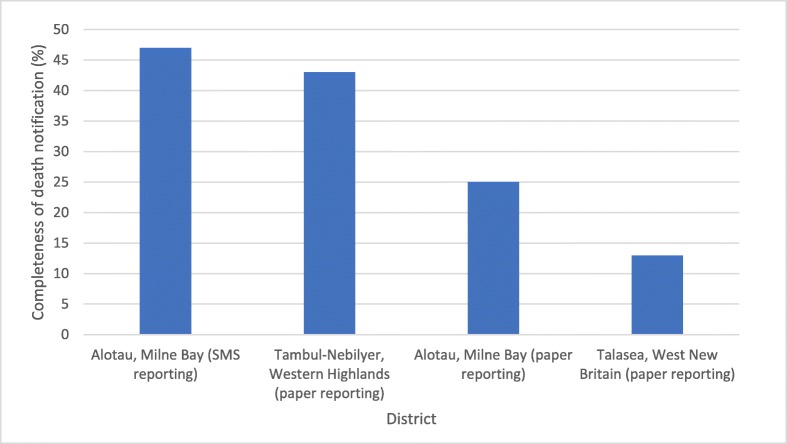
Percentage of estimated total deaths in the target districts notified to health centres, 2018, Papua New Guinea

Data collection took place between June 2018 and March 2019, and from a baseline of either no or very few out-of-facility deaths being notified in these areas, both paper and text methods demonstrated that death notification coverage could be increased to about one-third, on average^1^ (Fig. 3). While this improvement was not as great as expected, the procedures, skills and knowledge have now been established in these districts, and should significantly increase notification completeness over the next phase of implementation (2019–2021).

The evidence from PNG, arguably one of the most difficult countries in which to implement death notification systems due to difficult terrain, poverty and other factors, suggests that a mixed system, involving different notifying agents and notification techniques (paper based and SMS, ideally not charged to the sender), when combined with oversight and verification by the local health authorities, can significantly improve notification and COD information about community deaths.

## Conclusion

Notification of the fact of death is a critical first step in any vital registration system intended to provide the essential health intelligence for policy and planning. Notifications have languished globally, in part because of passive reporting procedures, but also because of a lack of innovation and research on potential cost-effective ways to capture information about deaths. The examples from countries reported in this paper demonstrate that active death notification systems adapted to country circumstances can lead to significant improvements in knowledge about community mortality and COD patterns using automated VA methods. Use of key notifiers with knowledge of their communities and integration of ICTs, where appropriate, can significantly aid this process. There has been a lack of focused methods for improving death notification, hampering efforts to improve registration completeness. Notification, for example, as a step in the overall registration process, is not described in detail in the Principles and Recommendations for Vital Statistics Systems from the United Nations Statistics Division, which focus instead on the designation and role of informants [22]; this is despite the enormous benefit that notification information can have for ministries of health and planning.

A key finding from the country examples reported here is that the move from a passive to an active notification system can have almost immediate benefits. In Bangladesh, strategic re-deployment of existing health staff highlights how targeted sensitisation and training programmes can effectively enhance notification from among existing community health workers. This has also been demonstrated in Myanmar, with the integration of the new notification system into existing health structures, particularly in rural areas, proving very successful. In some contexts, as seen in PNG where reporting of events is challenging, government services and agents may not always be able to identify and report deaths for the more remote areas, although the experience in PNG suggests that higher reporting of events can also be achieved through the use of a number of different reporting agents in these areas.

The application of ICT in these countries has generally been efficient and resulted in an increase in death notifications. However, the potential for success of such technologies can be limited by the underlying organisational structures and overall CRVS processes, as was observed in the MOVE-IT pilot study in Tanzania [23]. SMS-based notification can be a useful means to capture information about deaths in countries where the infrastructure to process the data is established. For example, in Colombia, mobile phone reporting not only led to an increase in the number of registered deaths, with a probable COD, but also broadened the type of informants that government civil registration agencies could use, leading to a more active reporting system and increased coverage.

Innovative strategies to improve notification systems for community deaths (and thus the quality of mortality data) to inform government decision-making needs to be an operational research priority in LMICs. The four country examples reported in this paper demonstrate that systems tailored to a country’s geography, existing CRVS system, and human and ICT resources can be effectively deployed and will generate improvements in mortality data almost immediately. Such improvements to death notification systems can, and should, be trialled in many other settings to improve country knowledge about mortality levels and patterns – an essential source of evidence for policy and monitoring of progress against health and development goals.

## Data Availability

Not applicable.

## Footnotes

1 Completeness of death notification was calculated as the number of deaths notified to health centres as a proportion of the total deaths during the collection period, estimated using population data from the 2011 census, crude death rates and proportion of non-hospital deaths by province [21]
